# A Lady with Severe Abdominal Pain Following a Zumba Dance Session: A Rare Presentation of Bochdalek Hernia

**DOI:** 10.7759/cureus.2427

**Published:** 2018-04-05

**Authors:** Abdul Rehman, Abdul Majeed Maliyakkal, Vamanjore A Naushad, Hisham Allam, Ahmed M Suliman

**Affiliations:** 1 Department of Medicine, Hamad Medical Corporation; 2 Department of Emergency Medicine, Hamad Medical Corporation; 3 Department of Surgery, Hamad Medical Corporation

**Keywords:** diaphragmatic hernia, diaphragmatic hernia, bochdalek hernia, gastric perforation, strangulation

## Abstract

A Bochdalek hernia is a congenital diaphragmatic hernia that results from a failure of closure of the pleuroperitoneal folds during embryologic development. While it is most often diagnosed in neonates and infants, Bochdalek hernias can rarely present in adulthood for the first time. We describe the case of a 42-year-old lady who presented with sudden onset of severe abdominal pain following a Zumba dance session. Her chest radiograph showed an elevated left hemi-diaphragm with visualization of a gastric bubble in the thorax. A computed tomography (CT) scan of the abdomen showed a defect in the left hemi-diaphragm with herniation of the stomach and abdominal viscera through the defect. The patient was taken for diagnostic laparoscopy, and the diaphragmatic defect was repaired with a synthetic mesh. Perioperatively, perforation of the anterior wall of the stomach was noted, and a diagnosis of Bochdalek hernia with gastric strangulation was made. This case demonstrates a rare presentation of Bochdalek hernia in an adult with strangulation and perforation of the stomach. Clinicians need to be aware of this rare but life-threatening clinical entity in order to secure a timely diagnosis and institute appropriate management.

## Introduction

Congenital diaphragmatic herniae (CDH) result from a failure of closure of the pleuroperitoneal folds during embryologic development. Vicente Bochdalek first described the posterolateral CDH in 1848 and since then, this variety of CDH is referred to as Bochdalek hernia [1]. Most cases of Bochdalek CDH are identified in neonates and infants, but in some cases, delayed presentation may occur in later childhood. First presentation of Bochdalek CDH in adulthood is extremely rare, and close to 250 such cases have been reported in the literature previously [2]. Here, we describe the case of a 42-year-old lady who presented with sudden onset of abdominal pain following a Zumba dance session and was found to have an obstructed Bochdalek CDH.

## Case presentation

A 42-year-old lady of South African origin without any significant past medical history presented to the emergency department with a complaint of sudden onset epigastric and left upper quadrant abdominal pain for the past few hours. She described the pain as sharp in character, severe in intensity, and continuous and radiating to the left shoulder and back. This pain started while she was working out in her regular Zumba dance session and had remained constant since then. The abdominal pain was associated with a sensation of dizziness and shortness of breath. She felt nauseated but did not have any vomiting. She denied any previous history of similar abdominal pain, altered bowel habits, abdominal distension, urinary complaints, or menstrual complaints. She did not report any history of palpitations, chest pain, cough, or fever. She was not taking any regular medications and did not have a history of any previous surgery. She had no significant past medical history. She denied any history of trauma.

**Figure 1:**
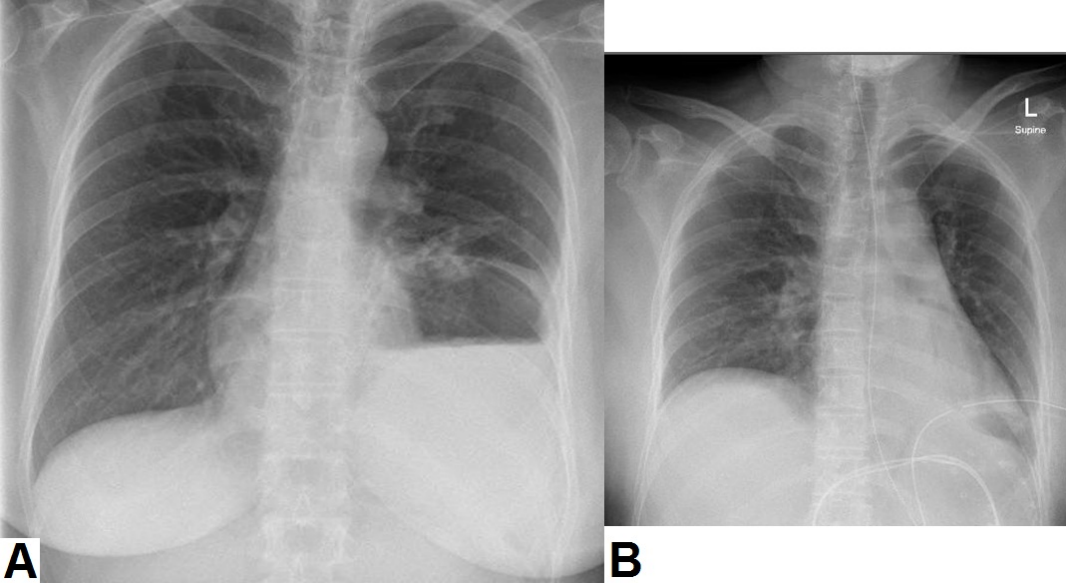
Plain chest radiographs of the patient (A) Plain chest radiograph showing a raised left hemi-diaphragm with visualization of a gastric bubble within the left hemi-thorax. (B) Postoperative radiograph demonstrates the presence of a surgical drain and nasogastric tube, and reduction in the level of the left hemi-diaphragm.

**Figure 2:**
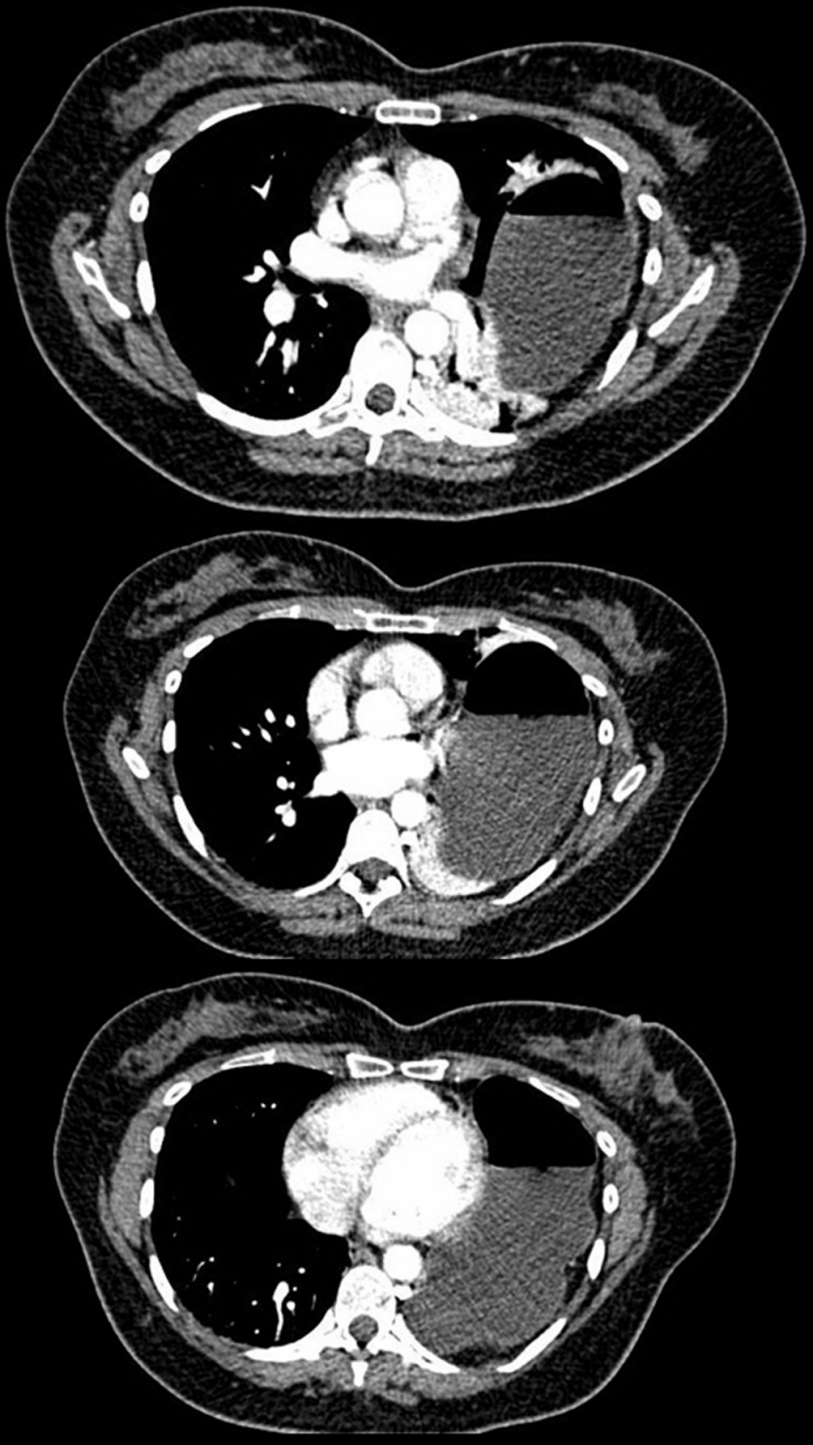
Thoracic computed tomography scans Axial sections of computed tomography scans demonstrate herniation of stomach into the left thoracic cavity.

Upon arrival to the emergency department, her vital signs were: pulse rate 96 beats per minute (regular), blood pressure 129/79 mm Hg (left arm), temperature 36.8°C (oral), and respiratory rate 19 breaths/min. On physical examination, abdominal examination revealed epigastric and left upper quadrant tenderness with guarding. No rebound tenderness or other peritoneal signs were noted. Bowel sounds were audible and normo-active. Laboratory investigations were within normal limits, except for a mild neutrophilic leukocytosis (white cell count of 13.6 × 109/L with 85% neutrophils). Serum beta-human chorionic gonadotropin (β-hCG) was within normal limits. Her plain chest radiograph (Figure 1-A) showed an elevated left hemi-diaphragm with visualization of a gastric bubble (fundus of the stomach) within the left hemi-thorax. Her electrocardiogram showed a normal sinus rhythm with no ST segment or T-wave abnormalities. A computed tomography (CT) scan of the chest, abdomen, and pelvis was performed, which revealed a defect in the posterolateral aspect of the left hemi-diaphragm with herniation of the entire stomach, spleen, tail of pancreas, and part of the splenic flexure into the thoracic cavity. These findings were concerning for an obstructed diaphragmatic hernia (Figure 2).

**Figure 3:**
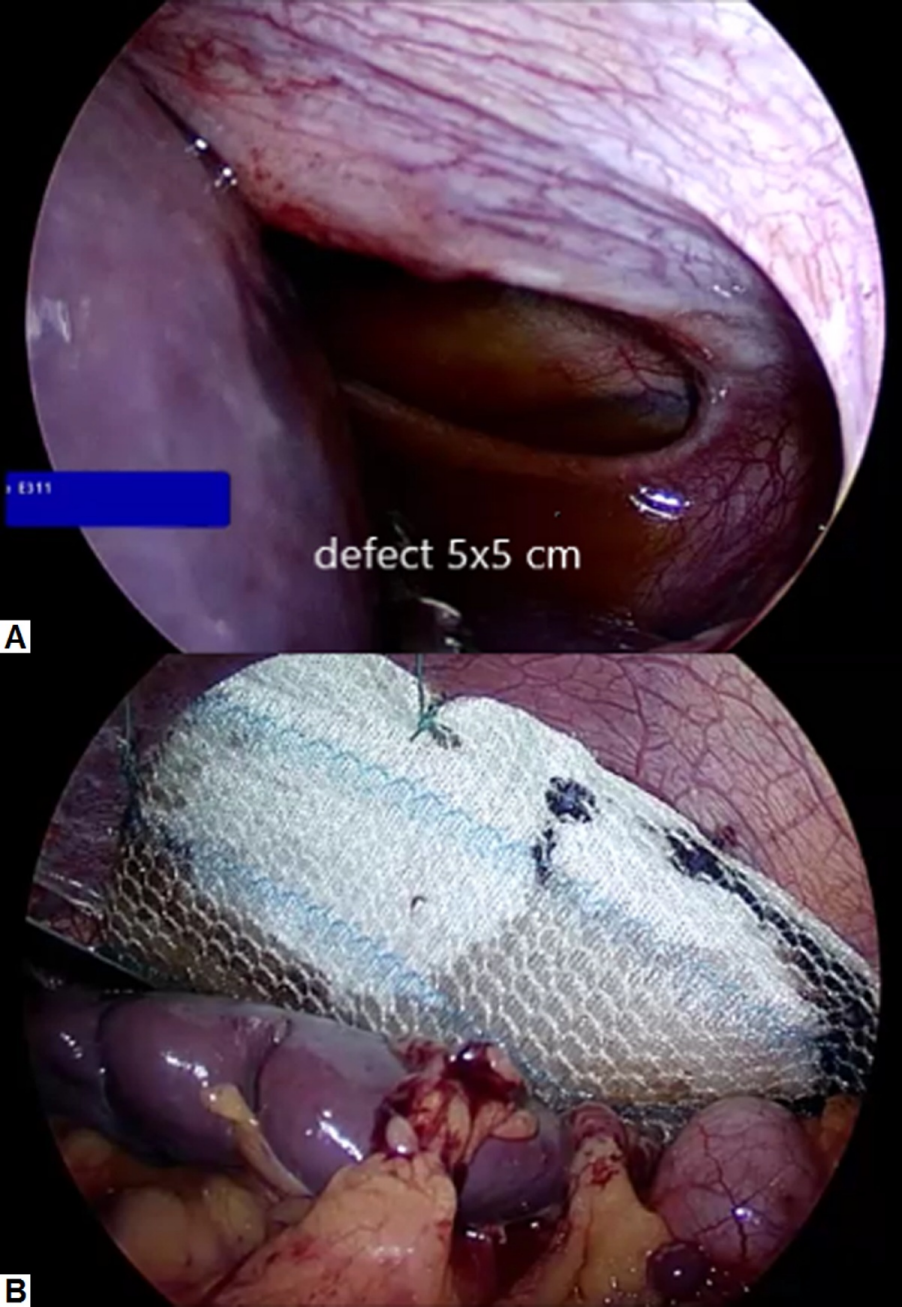
Laparoscopic pictures of the diaphragm (A) Intra-operative picture showing a large defect in the posterolateral aspect of the diaphragm. (B) The diaphragmatic defect has been repaired with the use of a synthetic biodegradable mesh.

The patient was kept nil by mouth and she was administered intravenous hydration and analgesia. A nasogastric tube was inserted for gastric decompression (after CT scanning had been performed). The general surgery team was consulted and the patient was taken to the operating room for diagnostic laparoscopy. Perioperatively, a large posterolateral Bochdalek hernia was identified with strangulation of the stomach. Abdominal viscera were reduced to the abdominal cavity and the diaphragmatic defect was closed (from below) using a synthetic biodegradable mesh (Gore Bio-A; W.L. Gore and Associates, Inc., Flagstaff, AZ) secured with non-absorbable sutures (see Figure 3). A small perforation was noted in the anterior wall of the stomach, which was repaired with absorbable sutures. Methylene blue was then injected through a nasogastric tube to ensure that there was no gastric leak. A Jackson-Pratt drain was left in place and abdominal port sites were closed. Postoperatively, the patient’s abdominal pain improved and she remained stable. Her postoperative chest radiograph is shown in Figure 1-B. Her drain was removed and she was discharged home after three days. She remained healthy at a one-month follow-up visit.

## Discussion

The diaphragm is a large skeletal muscle that separates the abdominal and thoracic cavities and serves as the chief muscle of respiration. Embryologically, development of the diaphragm starts during the third week of gestation from the mesodermal septum transversum. The septum transversum normally fuses with the dorsal mesentery of the foregut (esophagus), and the growth of the pleuroperitoneal membranes leads to the complete separation of the thoracic and abdominal cavities by the ninth week of gestation. Pleuroperitoneal hiatus are present in both sides of the septum transversum early in development, but both defects close around the eighth week of gestation. The left-sided defect is generally the last to close, and a failure of closure of this defect leads to a Bochdalek CDH [3]. This also explains why Bochdalek herniae tend to occur on the left side in almost 90% of cases. Herniation of abdominal contents—such as the stomach, liver, intestines, and spleen—can occur through the defect, and this can lead to pulmonary hypoplasia and respiratory distress.

Most cases of Bochdalek CDH are diagnosed in neonates or infancy and the incidence of this hernia has been reported to be approximately one in 4000 [4]. More cases of Bochdalek CDH occur in boys as compared to girls, with a male-to-female ratio of approximately 2:1 to 3:1. While most cases occur sporadically, clustering of Bochdalek CDH in certain families has also been reported [5]. The most common clinical presentation of Bochdalek CDH is respiratory distress in a neonate or infant. Infrequently, Bochdalek CDH may be diagnosed in early childhood or the first decade of life, either because of vague symptomatology or because it was identified incidentally on radiographs obtained for other purposes.

First presentation of a Bochdalek CDH in adulthood is exceedingly rare, and most such cases are diagnosed when the hernia becomes obstructed and complications develop [3-4]. Possible explanations for delayed presentation of Bochdalek CDH may include the small size of diaphragmatic defect, presence of a confining sac (made of pleura or peritoneum), and plugging of the defect with omentum. An acute increase in intra-abdominal pressure may lead to the sudden herniation of abdominal contents into the thoracic cavity, possibly precipitating incarceration. Previously reported cases have described a number of sequelae of Bochdalek CDH including gastric gangrene, ischemic bowel necrosis, splenic infarction, colopleural fistula, acute respiratory failure, and even sudden death [1-6]. In some previously reported cases, the occurrence of a gastric volvulus has been implicated in gastric strangulation [7]. Gastric volvulus is unlikely to occur in a normal stomach as it is well-suspended by the gastrohepatic, gastrocolic, gastrosplenic, and gastrophrenic ligaments. However, laxity or the absence of these ligaments in conjunction with CDH, a hiatal hernia, or diaphragmatic eventration may precipitate a gastric volvulus. In the present case, obstruction of the hernia with gastric strangulation and perforation was observed intra-operatively; no gastric volvulus was noted. A rise in intra-abdominal pressure has been attributed to a number of activities or precipitants in previously reported cases, such as trauma, physical exertion, sexual intercourse, sneezing and violent coughing, pregnancy and labor [1-7]. In one case, strangulation of a Bochdalek CDH was precipitated by the progressive growth of a large ovarian cyst. In the present case, physical exertion during the Zumba dance session may have led to an acute rise in intra-abdominal pressure, precipitating obstruction of the hernia.

A wide variety of clinical features have been reported in adult patients with Bochdalek CDH. The contents of the hernia can dictate the type of clinical symptoms that a patient may experience [2]. In left-sided herniae, herniated viscera may include the stomach, spleen, pancreas, transverse colon, jejunum, left lobe of liver, left suprarenal gland, and left kidney. On the right side, the liver, gallbladder, kidney, omentum, transverse colon, and jejunum may be found. In any case, hypoplasia and atelectasis of the lung is an invariable feature [3]. Most patients report vague chest tightness, abdominal discomfort, and a sensation of dyspnea (especially on exertion). Sudden onset of chest pain or abdominal pain along with nausea and vomiting is highly suggestive of obstruction and/or strangulation. In a series of 14 reported cases of right-sided Bochdalek CDH, 11 patients were noted to have visceral malformations in conjunction with the Bochdalek hernia, such as universal mesentery, intestinal maloration, gastric volvulus, and hepatic atrophy [8]. In our case, no such malformations were apparent.

The presence of a CDH can be readily detected on plain chest radiographs. However, a Bochdalek CDH can be confused for pulmonary sequestration, an epicardial fat pad, pneumonic consolidation, or left lower lobe collapse [2-4]. CT scans of chest and abdomen are generally more accurate in delineating the diaphragmatic defect and may also reveal signs of bowel ischemia or obstruction. The most common radiographic finding in a Bochdalek hernia is the presence of a fat or soft-tissue mass on the upper surface of the diaphragm along with a defect in the diaphragm adjacent to the mass [6]. CT scans are also superior to plain chest radiographs in differentiating Bochdalek CDHs from other types of CDH (e.g., Morgagni hernia). Other advanced imaging modalities, such as magnetic resonance imaging, also have comparable accuracy in delineating the hernia and the associated diaphragmatic defect. In the present case, the CT scan demonstrated a defect in the posterolateral aspect of the left hemi-diaphragm with herniation of the abdominal viscera, although no signs of visceral perforation were evident.

Traumatic diaphragmatic rupture is an important differential diagnosis in the evaluation of adult patients with a suspected Bochdalek CDH [6-7]. Although a history of trauma is an important clue to the presence of traumatic diaphragmatic rupture, CDH can be an incidental finding in patients with a history of trauma. Moreover, a history of trauma may not always be reported by patients, especially in cases of delayed presentation. In such cases, radiologic features may be useful in distinguishing between CDH and traumatic diaphragmatic rupture. Certain CT findings, such as the "collar sign" (constriction of the stomach as it passes through the diaphragmatic defect) and "dangling diaphragm sign" (visualization of the torn, free-edge of the diaphragm) are virtually pathognomonic of traumatic diaphragmatic rupture. Moreover, direct visualization of a diaphragmatic injury (discontinuity in the contour of diaphragm) and the "dependent viscera sign" (visualization of herniated viscera against the posterior chest wall) were also suggestive of a traumatic diaphragmatic rupture. Rarely, precise differentiation between traumatic diaphragmatic rupture and CDH may not be possible by radiologic means and an accurate diagnosis may only be made perioperatively.

Diagnosis of a Bochdalek CDH in an adult generally warrants surgical treatment as it is prone to obstruction and strangulation [4]. In the published literature, trans-thoracic and trans-abdominal approaches to repair a Bochdalek CDH have been described [9]. Repair of the diaphragm through a trans-thoracic approach (i.e., lateral thoracotomy) is easier as compared to trans-abdominal approach. However, in patients with signs of obstruction or strangulation, a trans-abdominal approach via laparoscopy or laparotomy is preferred. In the present case, the patient’s presentation was highly suggestive of obstruction and a laparoscopic exploration was performed. Minimally invasive procedures for repair of CDH are favored over invasive (open) procedures as the latter are associated with longer hospital stays and increased patient discomfort [10].

The use of mesh for repairing the diaphragm remains controversial and, in contrast with inguinal hernia repair, the superiority of tension-free diaphragmatic repair has not been demonstrated. Generally, elderly patients tend to have a thinner and weakened diaphragm, which makes the use of a mesh theoretically appealing. A variety of different surgical meshes are available, including non-absorbable prosthetic, acellular biological, and synthetic biodegradable meshes. To date, no clinical data have demonstrated the superiority of one type of surgical mesh over another. As biological meshes are costly and do not have any major advantages over synthetic biodegradable meshes, we chose to use the latter in the present case.

## Conclusions

Bochdalek CDH rarely presents in adulthood with signs of abdominal visceral obstruction and strangulation. Given that many such patients have vague clinical symptoms, a high index of clinical suspicion is necessary to secure a timely diagnosis and institute appropriate treatment. Clinicians in general, and emergency physicians in particular, need to be aware of this rare but life-threatening clinical entity.
